# Medical Syndicates

**Published:** 1890-11-22

**Authors:** 


### Medical Syndicates.

Within the last ten years there have been organized in France a considerable number of associations of physicians known as "*sindicats medicaux*." The main object in forming these associations has been to aid their members in obtaining proper remuneration for their professional services, by fixing a minimum scale of fees, by collecting and furnishing information with regard to bad paying patients, etc., and in general to provide the means of organized and systematic defence of the interests of the profession, and especially of the pecuniary interests of its members. The local syndicates have united to form departmental associations of like character—and they have an organ of publication, the *Concours Médical*, a weekly journal published in Paris. As an example we will take the syndicate of Morbiban, a sketch of the history of the organization of which with its constitution is given in the *Concours Médical* of the 21st June, 1890. The appeal sent to each physician in the department, sets forth that the medical profession is being invaded by persons who are illegally practising medicine that its interests are menaced by the organization and needs of co-operative societies, by the indifference of magistrates who permit empirics of all kind to practise, by the increase of religious orders, by pharmacists who repeat prescriptions without the order of the physician, and who even occasionally give medical advice, and by the fact that the Government and the great corporations do not pay enough for the services which they ask from medical men—and that the only remedy for this melancholy state of affairs is by organization of the doctors to oppose these sources of evil.

The statutes provide that any doctor of medicine or officier de santé (a physician of inferior qualifications) may become a member by subscribing to the regulations and paying ten francs a-year. The syndicate is divided into four groups, each group being managed by a syndic or chief, a secretary, and a treasurer, whose business it is to centralise the action of the members, to be in constant communication with the president of the syndicate, and to keep him informed of all irregularities of practice occurring in the district, and of the needs and desires of the members. The business of the syndicate is managed by an executive committee, composed of a president, the four chiefs of the groups, with a secretary and a treasurer, these officers being elected by the members for a term of three years.

The president is to receive, preserve, and use all documents addressed to the syndicate; the complaints, etc., are not to be signed, but are to be accompanied by a signed letter of transmittal, which is to be considered confidential. Thus any physician can present anonymous charges or complaints against any-one, and it is at the discretion of the president as to whether these shall be brought forward for action on them by the syndicate. Forms of bills and of dunning letters are given, and a scale of fees is to be fixed. A code of ethics is usually adopted, but not always.

These syndicates then, are to all intents and purposes, trade unions. Now, men whose chief or only reason for practising medicine is to make money, may, without hindrance, form such a trade union, and try and get as high a price as possible for the merchandise which they have to sell, provided they do not interfere with the business of other men, or seek, like the great American trusts of a year or two ago, to hold a monopoly in the article in which they deal.

Whether it is a wise and proper thing for a physician to join such an association is quite another matter, which can best be considered with reference to individual cases. Those who desire reputation, power, and offices which shall enable them to control to a certain extent their fellow-practitioners, and to pose as their representatives before the public, but who neither expect nor desire to obtain such reputation and office by making discoveries or advances in medicine, will, of course, see that a syndicate affords a peculiarly favourable field for their ambition. The men who desire to make their living by medical practice with as little exertion as possible, who do not wish to study or to teach, and who are content to part with a certain amount of individual liberty, and to have others think and decide for them in matters of medical politics, will also find peace and comfort in the syndicate, for a time at least. It is often a great convenience to have a definite scale of prices in the shape of a fee bill, to which an individual patient or the general public can be referred. The old-fashioned notion that the fee should be a sort of gratuitous offering from the patient does not work well in modern practice; but, on the other hand, it is very difficult to fix such a scale of prices which will not at times be unjust either to the patient or to the practitioner. To the young man struggling to build up a practice it seems unfair that he should be compelled to charge the rates which are acceptable to his seniors, who have acquired reputation, and whose waiting-rooms are crowded with patients. The fixing of minimum prices is the trade union scheme for levelling downward, but it is only efficacious when the union has a practical monopoly of the service to be rendered. The usual advice given by the elders to the juniors is—Don't practice medicine at reduced rates, for if you do your patients will esteem you at your own valuation, and your confrères will strongly disapprove. You may practice better than they do, if you can, but not more cheaply. The advice is good, but there always have been, and always will be, a vast number who do not follow it.

But if the struggle for sufficient remuneration to make the time and money spent in obtaining a medical education a paying investment is becoming arduous in France, what must it be elsewhere? In round numbers, France has about one person holding the diploma of doctor of medicine to each 3,000 of population, having 38,000,000 people, 12,000 physicians, and graduating about 625 medical students a-year.

Germany has about 45,000,000 people, 30,000 physicians, and graduates 935 students per year, and some of its philosophers are beginning to devise ways and means of checking the overcrowding. The United States has about 63,000,000 of population, 90,000 physicians, and graduates over 3,000 new ones every year; and, under these circumstances, it is probable that over half of them cannot pay their expenses from the results of their practice alone, if they live in such a manner as to make existence desirable. The deficiency must be supplied by inherited capital and from parents and friends, marrying money, or by joining some other occupation to that of the practice of medicine.

Limitation by law of the increase in the number of physicians is both undesirable and impossible. That State regulation of the amount of knowledge which must be shown before permission to practice is given does limit the number of aspirants somewhat is true; but such regulations are not made for the benefit of the medical profession, and are only justifiable for the purpose of protecting the health and lives of the people.

There is, of course, quite another aspect of medicine from that presented by its trade side. It has been compared to the moon, one side of which is always in shadow, while the other is more or less illuminated at different times.

There are many men who have studied, and who now practice, medicine with little thought as to the money to be thus made, and there are a vast number who, though dependent on it for their daily bread, would not abandon it for a more lucrative occupation, because they find their greatest enjoyments in the work itself. To all such men the syndicate view of things will be more or less repugnant; nor will they be willing to join in a socialistic movement which requires them to give up a certain amount of individual liberty, and to sustain their political leaders in their efforts to obtain higher wages.

